# The Effect of Allulose on the Attenuation of Glucose Release from Rice in a Static In Vitro Digestion Model

**DOI:** 10.3390/foods13152308

**Published:** 2024-07-23

**Authors:** Leila Hammond, Megan Wurtele, Ricardo de Almeida, Constança Silva, Janine DeBlasi, Yan Lu, Nick Bellissimo

**Affiliations:** 1 School of Nutrition, Toronto Metropolitan University, Toronto, ON M5B 2K3, Canada; 2 Department of Chemistry and Biology, Toronto Metropolitan University, Toronto, ON M5B 2K3, Canada; 3 Department of Molecular Pharmacology and Physiology, Morsani College of Medicine, University of South Florida, Tampa, FL 33620, USA; 4 Heilongjiang Green Food Science Research Institute, Harbin 150086, China

**Keywords:** allulose, fructose, rare sugars, in vitro digestion, starch digestibility

## Abstract

Allulose is a rare sugar that provides <10% of the energy but 70% of the sweetness of sucrose. Allulose has been shown to attenuate glycemic responses to carbohydrate-containing foods in vivo. This study aimed to determine the optimal allulose dose for minimizing in vitro glucose release from rice compared to a rice control and fructose. A triphasic static in vitro digestion method was used to evaluate the in vitro digestion of a rice control compared to the co-digestion of rice with allulose (10 g, 20 g, and 40 g) and fructose (40 g). In vitro glucose release was affected by treatment (*p* < 0.001), time (*p* < 0.001), and treatment-by-time interaction (*p* = 0.002). Allulose (40 g) resulted in a reduction in in vitro glucose release from rice alone and rice digested with allulose (10 g), allulose (20 g), and fructose. The incremental area under the curve (iAUC) for in vitro glucose release was lower after allulose (40 g) (*p* = 0.005) compared to rice control and allulose (10 g) but did not differ from allulose (20 g) or fructose. This study demonstrates that allulose reduces glucose release from carbohydrates, particularly at higher doses, underscoring its potential as a food ingredient with functional benefits.

## 1. Introduction

The deleterious effects of added sugars, in particular fructose sweeteners, on human health have become a public health concern [1,2]. Chronic fructose consumption has been attributed to the increasing prevalence of obesity and chronic health conditions, including metabolic syndrome [2]. This has led to an increased interest in fructose and sucrose alternatives and understanding their metabolic effects, particularly regarding their impact on glycemic response and glucose metabolism [3,4]. Rare sugars are monosaccharides or disaccharides that occur naturally in food in relatively small quantities [5,6].

Allulose (D-Psicose), the C-3 epimer of D-fructose, is a rare monosaccharide that provides <0.2–0.4 kcal/g while having 70% of the sweetness of sucrose [5,7,8]. In the United States, allulose has been classified by the Food and Drug Administration as generally recognized as safe [9]. Several animal and human studies have found that allulose can attenuate the glycemic response to carbohydrate-containing meals [10,11,12,13,14]. A systematic review evaluating the effects of fructose and its epimers on postprandial glycemic response in humans concluded that allulose reduced blood glucose postprandially and led to improvements in insulin regulation compared to fructose [12]. This property of allulose positions it as a unique functional food ingredient due to its potential ability to attenuate glycemic response. 

Despite differences in how the human body metabolizes allulose compared to its epimer, fructose, both follow similar absorption pathways due to similarities in their structures [15]. There are several proposed mechanisms for the effects of allulose on postprandial glycemic response. These include the inhibition of amylase enzymes throughout the digestive tract, the inhibition of glucose transporters (GLUT) in the intestine, the stimulation of hepatic glycogenesis, and the modulation of postprandial hormones with glucoregulatory effects, such as glucagon-like peptide-1 [16,17].

In vitro methods for the determination of carbohydrate content in foods were first established in the 1960s [18]. Several in vitro methods have been developed that serve as models for simulating human digestion and the glycemic responses of carbohydrate-containing foods [19,20,21]. In particular, in vitro starch digestion has been shown to be highly correlated with in vivo trials [20]. Although in vitro digestion models are not able to replicate all dynamic aspects of the gastrointestinal tract, they offer a more cost-effective, faster, and convenient method of determining proxy glycemic responses to foods without a high level of inter- and intra-individual variability, which are often limitations of glycemic response studies in humans [20,21]. 

Considering the unique sensory and metabolic properties of allulose, the objective of the present study was to evaluate the dose–response effect of allulose digested with a high-glycemic carbohydrate on in vitro glucose release compared to its epimer, fructose, and a carbohydrate control.

## 2. Materials and Methods

### 2.1. Experimental Design

A static triphasic in vitro digestion method, Dedicated Ryerson University In-Vitro Digester (DRUID) [22], that replicates the oral, gastric, and small intestinal phases of human digestion was used to determine in vitro glucose release in response to varying treatments. Differing doses of allulose (10 g, 20 g, and 40 g) were compared to fructose and white rice alone (carbohydrate control) to evaluate the in vitro release of glucose. The optimal dose of allulose for attenuating in vitro glucose release was used to select the dosage for fructose. In vitro glucose release was measured every 15 min in the intestinal phase over 120 min. 

### 2.2. Materials

Hydrochloric acid (12N) was obtained from Caledon Laboratories Ltd. (Georgetown, ON, Canada). Sodium hydroxide (pellets), potassium chloride (crystalline), sodium bicarbonate (crystalline), and sodium chloride (crystalline) were obtained from Fischer Scientific (Ottawa, ON, Canada). α-Amylase from Bacillus sp. (A3404), pepsin from porcine gastric mucosa (P7125), amyloglucosidase from Aspergillus niger (A7095), and pancreatin from porcine gastric mucosa (P7000) were obtained from MilliporeSigma Canada (Oakville, ON, Canada). 

Instant rice (Minute Rice Premium Instant Long Grain White Rice, Riviana Foods, Houston, TX, USA) was obtained from a local market. Allulose (allSWEET^®^ Crystalline Powder) was obtained from Anderson Advanced Ingredients (Irvine, CA, USA). Fructose Crystalline (>99.5%) was obtained from Ingredient Depot (Beauharnois, QC, Canada). 

### 2.3. Sample Preparation

#### 2.3.1. Test Foods

Each serving of white rice provided 50 g of available carbohydrates, 5.3 g of protein, and 0.4 g of fat, which was determined using the nutrition facts table provided by the manufacturer. The white rice was prepared by cooking 61.0 g of rice in 165 mL of tap water [23] in a rice cooker (3-cup rice cooker, Black & Decker Corporation, Beachwood, OH, USA) for 12 min according to manufacturer directions. The rice was left to cool down for approximately 5 min until the steam was no longer visible.

#### 2.3.2. In Vitro Digestion Solutions

The in vitro digestion model used was adapted from the INFOGEST protocol [24] and was validated in vivo [25].

##### Simulated Saliva

Artificial saliva was prepared by combining 0.117 g of sodium chloride, 0.149 g of potassium chloride, and 2.1g of sodium bicarbonate into 1000 mL of ultrapure water (UPW) (Direct-Q^®^ 3 UV Remote Water Purification System, MilliporeSigma, Oakville, ON, Canada). The final artificial saliva solution was 2 mM sodium chloride, 2 mM potassium chloride, and 25 mM sodium bicarbonate. 

##### Simulated Gastric Solution

The simulated gastric fluid was prepared by dissolving 2.5 g of pepsin in a 50 mL beaker containing 23.75 mL of UPW and 1.25 mL of 1 M hydrochloric acid (HCl). The solution was stirred with a magnetic stirrer at 500 RPM for 5 min. 

##### Simulated Pancreatic Solution

The simulated pancreatic solution was prepared by dissolving 2.5 g of pancreatin in 100 mL of UPW. The solution was stirred with a magnetic stirrer at 500 RPM for 15 min.

### 2.4. Static In Vitro Digestion

A triphasic static in vitro digestion method that replicates the oral, gastric, and small intestinal phases of human digestion was used [22]. Each in vitro digestion trial was performed in duplicate for each treatment. 

#### 2.4.1. Oral Phase

To begin the oral phase, simulated mastication was performed by adding the rice into a commercially available blender (Ninja BL611C Professional Blender, SharkNinja Operating LLC, Needham, MA, USA). The rice was blended on the “medium” setting (3600–4600 RPM) for 10 s to create a thick, lumpy paste-like consistency with some remaining intact rice grains. The blended rice sample was then added to a 3 L glass bioreactor (HyPerforma, Thermo Scientific, Waltham, MA, USA), followed by the test treatments. The bioreactor was then transferred to a water bath and kept at 37 °C for the remainder of the experiment.

For every 100 g of test food, 20 mL of artificial saliva was added. UPW was added so that the total volume of fluid added to the vessel was 300 mL. An electric overhead stirrer fitted with a dual 4-blade impeller was added to the bioreactor, which stirred at a constant rate of 60 RPM for the remainder of the experiment. Then, 2 mL of α-amylase was added to the bioreactor, and the oral phase continued for 2 min. 

#### 2.4.2. Gastric Phase

After the oral phase incubation, 5–12 mL of 1 M HCl was added to the same bioreactor to bring the pH of the solution to 1.75–2.00. The pH was measured via a digital pH Meter (OHAUS pH Meter Starter 2200 Bench, Mettler-Toledo International Inc., Columbus, OH, USA). Once the desired pH was achieved, 10 mL of the simulated gastric fluid was added to the bioreactor, marking the beginning of the gastric phase. During the 30 min of gastric digestion, aliquots of 1 mL were taken from the bioreactor at 15 and 30 min to assess in vitro gastric glucose release. Aliquots were taken at each timepoint by collecting ~0.33 mL from three different locations of the bioreactor using a 1 mL pipette. The 1 mL aliquot was diluted into 8 mL of UPW in a polystyrene conical tube (15 mL Falcon, Corning Inc, Corning, NY, USA) and placed in an ice bath for at least 5 min before glucose determination. The glucose value obtained at 30 min of gastric digestion was reported as the baseline (0 min) timepoint in the statistical analysis.

#### 2.4.3. Intestinal Phase

After 30 min of gastric incubation, sodium hydroxide was added to the same bioreactor to bring the pH to 5.85–6.15. Then, 100 mL of simulated pancreatic solution and 2 mL of amyloglucosidase were added to the bioreactor, and the content in the bioreactor was mixed at a constant rate of 60 RPM for 120 min. Aliquots were taken from the bioreactor in 15 min intervals from 15 to 120 min of the intestinal phase.

### 2.5. Glucose Concentration Determination

Diluted aliquots were removed from the ice bath and mixed on a benchtop vortex mixer (Benchmixer™ Vortexer Mixer, Benchmark Scientific, Sayreville, NJ, USA) at 3200 RPM for 10 s. The aliquots were then centrifuged at 5000 RPM for 5 min at 4 °C (Eppendorf 5804R Refrigerated Centrifuge, Eppendorf Canada, Mississauga, ON, Canada). The supernatant was then analyzed in duplicate for glucose concentration using a biochemistry analyzer (YSI 2950D, YSI Incorporated, Yellow Springs, OH, USA).

### 2.6. Statistical Analysis

The trapezoid method was used to calculate the incremental area under the curve (iAUC) [26,27]. Comparisons of iAUC values were performed using a one-way analysis of variance (ANOVA). A two-way ANOVA was performed to evaluate the treatment-by-time interaction. A Tukey post hoc analysis was performed to determine differences among treatments. Statistical significance was considered as *p* < 0.05. All analyses were conducted using GraphPad Prism Version 10.2.2.

## 3. Results

### 3.1. In Vitro Glucose Release Over Time

**Figure 1:**
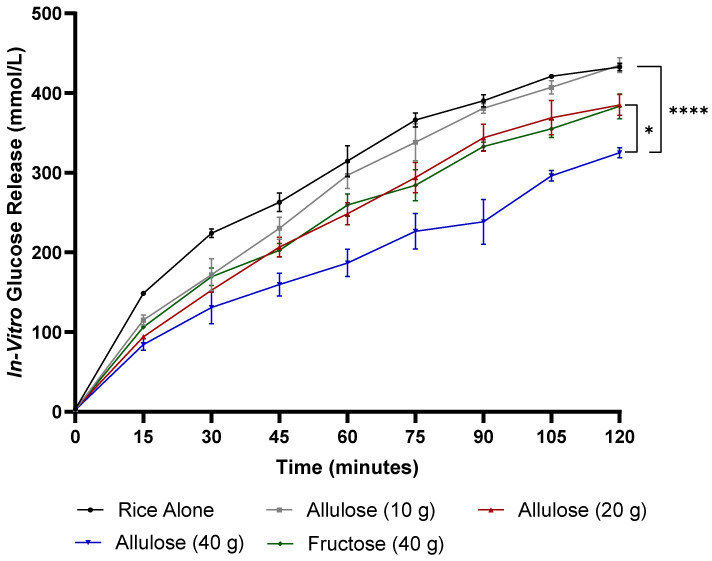
The in vitro digestion of differing doses of allulose compared to fructose and rice alone: in vitro glucose release (mmol/L) over 120 min. Error bars represent the standard error of the mean (SEM) (*n* = 2). Statistical significance is set at *p* < 0.05. * *p* < 0.05, **** *p* < 0.0001 (two-way ANOVA with Tukey’s post hoc analysis).

In vitro glucose release during the intestinal phase was affected by treatment (*p* < 0.001), time (*p* < 0.001), and treatment-by-time interaction (*p* = 0.002) (Figure 1). In vitro glucose release was lower after 40 g of allulose compared to rice alone at 15, 30, 45, 60, 75, 90, 105, and 120 min (*p* < 0.01). In vitro glucose release was lower following fructose compared to rice alone at 30, 45, 60, 75, 90, and 105 min (*p* < 0.05). In vitro glucose release was lower after 40 g of allulose compared to 10 g of allulose at 45, 60, 75, 90, 105, and 120 min (*p* < 0.01). In vitro glucose release was lower with 40 g of allulose compared to 20 g of allulose at 60, 75, 90, 105, and 120 min (*p* < 0.05). In vitro glucose release was lower following 40 g of allulose compared to fructose at 60, 75, 90, 105, and 120 min (*p* < 0.05). 

In vitro glucose release was lower with 20 g of allulose when compared to rice alone at 15, 30, 45, 60, and 75 min (*p* < 0.05) and following 10 g of allulose compared to rice alone at 30 min (*p* < 0.05). Fructose resulted in significantly lower glucose release in vitro when compared to 10 g allulose at 75 min (*p* < 0.05). In vitro glucose release did not differ between 20 g of allulose when compared to fructose.

### 3.2. The Incremental Area under the Curve Glucose Release (iAUCGR)

**Figure 2:**
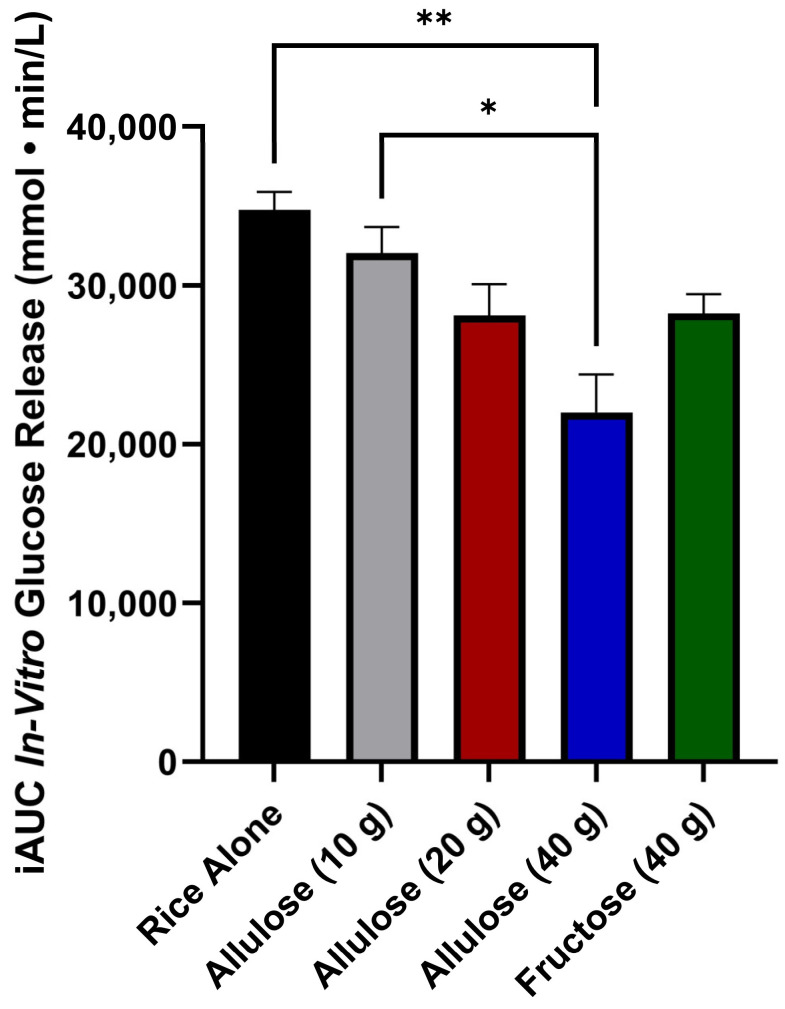
The iAUC in vitro glucose release (mmol·min/L) over 120 min; values are reported as the incremental area under the curve (iAUC). Error bars represent the standard error of the mean (SEM) (*n* = 2). Percent decrease from rice alone was 7.9% (allulose, 10 g), 19.1% (allulose, 20 g), 36.7% (allulose, 40 g), and 18.8% (fructose, 40 g). Statistical significance is set at *p* < 0.05. * *p* < 0.05, ** *p* < 0.01 (one-way ANOVA with Tukey’s post hoc analysis).

The iAUCGR was affected by treatment (*p* = 0.005) (Figure 2). The iAUCGR was higher for rice alone compared to rice digested with 40 g of allulose (*p* < 0.01) but not 20 g allulose (*p* = 0.06), 10 g of allulose (*p* = 0.56), or fructose (*p* = 0.06). The iAUCGR of rice digested with 10 g of allulose was higher compared to 40 g allulose (*p* < 0.05) but did not differ from 20 g of allulose (*p* = 0.29) or fructose (*p* = 0.31). The iAUCGR did not differ when fructose was compared to 20 g of allulose (*p* = 1) or 40 g of allulose (*p* = 0.07). There was no significant difference between the iAUCGR of 20 g allulose and 40 g allulose (*p* = 0.08).

## 4. Discussion

This study is the first to compare the effects of different doses of allulose on the release of glucose from starch in an in vitro digestion model. The findings of this study demonstrate a dose-dependent effect of allulose on glucose release from rice. Indeed, the highest dose of allulose evaluated (40 g), which approximates the available carbohydrates (50 g) provided by the rice, yielded a significant reduction in in vitro glucose release. Although the effect of allulose is dose-dependent, the decrease in the iAUCGR from rice alone for allulose (10 g), allulose (20 g), and allulose (40 g) was 7.9%, 19.1%, and 36.7%, respectively. 

Previous investigations in animals and humans have found that allulose, when consumed with a carbohydrate-containing meal, can attenuate the glycemic response. Allulose has been shown to reduce the glycemic response to a carbohydrate load in rats [13]. In humans, acute clinical trials demonstrate that allulose consistently reduces the glycemic response to carbohydrate-containing test meals [14]. The present study found that rice digested with allulose, particularly with 40 g of allulose, had a lower iAUCGR compared to rice alone. In alignment with the results of the present study, research to date shows that higher doses of allulose result in greater attenuations of glycemic response, although none of the studies that have evaluated the dose effects of allulose had a starch-based carbohydrate load [10,11,28,29]. Animal and clinical trials that have examined the effects of allulose on glycemic responses have used lower doses than those used in the present in vitro study [10,11,28,29]. To the best of our knowledge, the highest dose of allulose used in a human study evaluating glycemic response was 15 g, which corresponded to 50% of the weight of the carbohydrate given [10]. Indeed, the highest dose used in the present study likely exceeds the suggested upper limit of allulose (0.4 g/kg of body weight), which may increase the risk of gastrointestinal symptoms in some individuals [30]. 

Despite the evidence demonstrating the ability of allulose to reduce postprandial glycemia, the underlying mechanism remains subject to ongoing debate, reflecting the complexities involved in understanding its physiological effects. There are several hypotheses about the mechanism [16]; however, given the in vitro digestion model used in the present study, any attenuation of in vitro glucose release from starch is likely due to the suppression of digestive enzymes or interactions of allulose and the rice starch. 

A review of previous research suggests that allulose inhibits salivary and intestinal α-amylase, sucrase, and maltase [13]. It has been proposed that allulose may have inhibitory effects on pancreatic α-amylase and amyloglucosidase due to allulose having similar known inhibitory functions as acarbose [16]. These enzymes play crucial roles in breaking down complex carbohydrates, such as starch, into simpler sugars like glucose [16]. It has been suggested that allulose inhibits the activity of the aforementioned enzymes, leading to a reduced rate of starch digestion and subsequent glucose release [13]. By impeding the breakdown of starch into glucose, allulose contributes to blunted postprandial glycemic responses, offering potential benefits for managing blood glucose levels and metabolic health [16]. The enzymes in the in vitro digestion process used in the present study were bacterial α-amylase, pancreatic trypsin, amylase and lipase, ribonuclease, protease, and amyloglucosidase. Amyloglucosidase, acting as a key starch-digesting enzyme in the in vitro method employed in our study, plays a crucial role in catalyzing the hydrolysis of α-1,4-glycossidic linkages in starch molecules. Consequently, the outcomes of our study hold potential to further validate the hypothesis that the enzymatic inhibitory properties of allulose extend to amyloglucosidase. Moreover, by elucidating how allulose impacts amyloglucosidase activity, our findings can provide valuable insights into the mechanism underlying the observed reductions in glycemic response associated with allulose consumption.

While the present study found that allulose decreased rice starch digestion into glucose, research into the effects of allulose on the physical properties of starch shows mixed results on its impact on starch retrogradation and gelatinization [31,32,33]. Some findings suggest that allulose interacts with the starch matrix in foods, affecting its physical properties such as retrogradation and gelatinization [32,33], and these interactions can potentially alter starch digestion and glucose release [34]. For example, lower starch gelatinization, retrograded starch, and a high amylose content have been shown to reduce postprandial glycemia [35]. In the present study, instant long-grain rice was used and parboiled. This type of rice has been associated with having increased resistant starch content [36], a higher amylose content, and a reduced glycemic response compared to other rice varieties [37]. Since the rice portion remained consistent across all test conditions, the reduction in postprandial glycemic response is likely attributed to the effect of the test treatments. Given our emphasis on assessing the dose–response effect of allulose on in vitro glucose release relative to fructose and a carbohydrate control, it is pertinent to acknowledge the nuanced findings regarding the aforementioned interaction. While some studies suggest potential interference with starch retrogradation others present varied outcomes regarding gelatinization [31,32,33]. Such insights could provide a more comprehensive understanding of how allulose influences the digestion dynamics of rice and subsequent glucose release. 

In addition to evaluating various doses of allulose, this study conducted a comparative analysis between allulose and fructose to assess their respective impacts on in vitro glucose release from rice. This study found that allulose, particularly at higher doses, exhibited a more pronounced inhibitory effect on in vitro glucose release during digestion compared to fructose. In the present study, fructose and allulose (40 g) differed significantly in in vitro glucose release when digested with rice beginning at 60 min into the intestinal phase until the end of the experiment (120 min). These findings partially support much of the animal and human research to date. A recent systematic review of fructose and its epimers (allulose and tagatose) found that fructose had a limited effect on postprandial glycemia, whereas allulose decreases postprandial blood glucose levels [12]. Interestingly, there have been no studies that have compared allulose and fructose to a negative control. The current study supports the hypothesis that some of the differences seen between the glycemic effect of fructose compared to allulose may be related to enzymatic inhibition during the digestive process. 

The metabolic differences between allulose and fructose, arising from their distinct structural configurations, are notably influenced by enzymatic transformations [38] and may offer another insight into the observed findings of the present study. The conversion of fructose to allulose, catalyzed by enzymes such as D-allulose 3-epimerases, induces a pivotal structural alteration at the C3 position [38]. Enzymatic modification significantly alters their respective metabolic pathways despite both sugars sharing analogous absorption mechanisms [38]. One study found that allulose demonstrated metabolic differences compared to fructose, particularly in terms of metabolic stability within human and rat hepatocytes [39]. In the same study, allulose demonstrated remarkable stability over a 240 min period, retaining between 94.5% and 96.8% of its initial concentration [39]. In contrast, fructose underwent rapid metabolism, with only 43.1% to 52.6% remaining within the same timeframe [39]. This divergence suggests distinct metabolic pathways for these two sugars, although absorption mechanisms remain similar. This aligns with our data, which found a greater effect of allulose (40 g, 36.7%) compared with the same dose of fructose (40 g, 18.8%). Following absorption through facilitated diffusion, utilizing transporters such as GLUT5 for fructose and potentially GLUT2 and GLUT5 for allulose within the small intestine, both sugars enter the systemic circulation [16,40]. However, their divergent metabolic fates post-absorption significantly impact physiological responses and health outcomes [38], though our findings found no divergence between allulose and fructose in vitro glucose release until 60 min into the intestinal phase. 

Fructose undergoes hepatic metabolism via phosphorylation by fructokinase, initiating the generation of triose phosphates, which can be further metabolized into either glycogen or lipids [41]. Conversely, the unique structural configuration of allulose limits its metabolic fate. Despite its monosaccharide classification, allulose exhibits minimal metabolism within the body [38]. These structural differences render allulose predominantly excreted unchanged in the urine, thereby contributing negligibly to overall caloric intake. It has been hypothesized that this property of allulose may serve as a substrate for microbes in in vivo trials [42,43], although further research is required and is outside the purview of the in vitro digestion model used in the present study. Acknowledging the distinct metabolic properties of allulose highlighted in previous research is essential for understanding its potential as a sugar alternative with health benefits, particularly for weight management and glycemic control. By delineating the metabolic differences between allulose and fructose, our study can contribute valuable insights into their physiological impacts on glucose release from rice in a static in vitro digestion model, informing potential dietary interventions aimed at optimizing health outcomes.

Despite the contributions of this study to the understanding of the influence of fructose and allulose on carbohydrate digestion, its findings must be interpreted in the context of the following limitations. While the digestion model used provides a reproducible way to mimic human digestion, the simplicity of the static in vitro digestion model used may limit the applicability of these results to what would be seen in humans [20]. The mechanisms by which fructose and sucrose alternatives affect postprandial glycemia are multifactorial, and the in vitro methods used in this study can only capture the effects on enzymatic digestion [16]. Future in vivo studies comparing the effects of allulose to fructose are required to obtain a comprehensive understanding of these effects.

## 5. Conclusions

When digested in combination with white rice, allulose significantly reduces the in vitro release of glucose from rice starch. The extent of this effect is dose-dependent, where the highest dose of allulose resulted in a significant reduction in in vitro glucose released from a similar amount of rice. Furthermore, only half the amount of allulose (20 g) was required to produce a similar glycemic response compared to fructose (40 g). This positions allulose as a promising functional food ingredient that reduces the calories and carbohydrates of foods not only through the substitution of fructose but also through attenuated carbohydrate digestion, which may have public health implications and additional applications for glycemic control. 

## Data Availability

The original contributions presented in the study are included in the article, further inquiries can be directed to the corresponding author.
